# Pharmacometric analyses and clinical evidence for brigatinib dosing in anaplastic lymphoma kinase‐positive non‐small cell lung cancer

**DOI:** 10.1111/bcp.15322

**Published:** 2022-03-29

**Authors:** Neeraj Gupta, Michael J. Hanley, Vikram Sinha

**Affiliations:** ^1^ Takeda Development Center Americas, Inc. Lexington Massachusetts USA

We read with interest the study by Koele and colleagues^1^ in which the authors conclude that a more favourable benefit–risk profile of brigatinib can be achieved through a pharmacokinetically (PK) guided precision‐dosing strategy. According to their analysis, the optimal predicted maintenance dose is 305 mg QD, an approximately 70% increase over the currently approved 180 mg QD dosing regimen. In a phase 1/2 study, doses of 300 mg QD demonstrated dose‐limiting toxicity (grade 4 dyspnoea) relative to lower doses.^2^ The selection of brigatinib 180 mg QD with a 7‐day lead‐in at 90 mg QD (90 → 180 mg QD) for phase 2 and phase 3 studies was in part based on the absence of dose‐limiting toxicities at 180 mg QD in the phase 1/2 study.^2^, ^3^, ^4^ Subsequent exposure‐response analyses demonstrated that a 180 mg QD dose provides systemic exposures of brigatinib associated with high efficacy while maintaining an acceptable safety profile.^5^ Furthermore, retrospective analyses of real‐world expanded access program and claims data showed that brigatinib provided durable efficacy and was associated with high (92.7%) adherence to treatment in patients with heavily pretreated advanced anaplastic lymphoma kinase‐positive non‐small cell lung cancer (*ALK+* NSCLC) in clinical practice.^6^, ^7^, ^8^, ^9^ Finally, it should be noted that brigatinib is not administered as one fixed dose; the recommended dosing of 90 mg QD for 7 days followed by 180 mg QD ensures a favourable benefit–risk profile compared with administration of a 90 mg or 180 mg fixed dose.^2^, ^3^

Our dose optimization analyses are described in the paper “Brigatinib Dose Rationale in Anaplastic Lymphoma Kinase–Positive Non‐Small Cell Lung Cancer: Exposure‐Response Analyses of Pivotal ALTA Study.”^5^ The benefit–risk modelling described in our paper is in agreement with Koele et al.^1^ regarding the positive relationship between dosage and efficacy in fixed‐dose scenarios. However, we suggest that Koele's conclusion of improved benefit–risk due to therapeutic drug monitoring (TDM) scenarios is not fully supported by the data.

We note that the efficacy improvements associated with TDM by the authors are less pronounced when comparing fixed‐dose scenario 1 (90 → 180 mg QD) with TDM scenario 1 (90 → 180 mg QD, followed by PK‐guided dose adjustments to reach the median trough concentration associated with 180 mg QD at steady state). Specifically, TDM scenario 1 resulted in only a 0.3‐month improvement in median progression‐free survival (PFS) (14.9 *vs*. 14.6 months) and a 1.8‐month improvement in median overall survival (OS) (51.2 *vs*. 49.4 months) compared with fixed‐dose scenario 1. Similar numerically small improvements in median PFS (15.8 *vs*. 16.2 months) and OS (55.3 *vs*. 57.2 months) were observed when comparing fixed‐dose scenario 2 (90 → 240 mg QD) with TDM scenario 2 (90 → 240 mg QD, followed by PK‐guided dose adjustments to reach the median trough concentration associated with 240 mg QD at steady state). Accordingly, we believe it can be argued that this in silico study by Koele et al. provides support for the currently approved fixed‐dosing approach, as TDM was predicted to result in numerically small increases in efficacy and would introduce additional burdens for both patients and the healthcare system through periodic blood draws and subsequent dose adjustments. We also note that the authors have not reported the number of patients predicted to require dose adjustment, which is essential in order to assess the veracity of such a model, nor specifically how dose escalation was achieved in the PK‐guided simulations (e.g., immediate escalation to the higher dose or via a step‐wise increase based on tolerability). These details are pertinent given that the authors note that, based on their simulations, patients may require doses of 360 mg, 450 mg, 600 mg, 900 mg, or 1800 mg. We are not aware of any evidence to suggest that doses 10‐fold higher than the currently approved 180 mg dose would result in sufficient tolerability for long‐term treatment.

Furthermore, our clinical efficacy and safety experience and exposure‐response analyses do not support modification of the recommended 180 mg QD (with a 7‐day lead‐in at 90 mg QD) brigatinib dosing regimen for patients with *ALK*+ NSCLC.^3^, ^4^, ^5^, ^10^ We therefore believe that the authors have not provided sufficient evidence to modify the current brigatinib dosing recommendations that were established through controlled clinical trials designed to optimize dose selection based on benefit–risk considerations.

## COMPETING INTERESTS

NG, MJH, and VS are employees of Takeda Development Center Americas, Inc.
